# ZipA and FtsA* stabilize FtsZ-GDP miniring structures

**DOI:** 10.1038/s41598-017-03983-4

**Published:** 2017-06-16

**Authors:** Yaodong Chen, Haiyan Huang, Masaki Osawa, Harold P. Erickson

**Affiliations:** 1 Key Laboratory of Resources Biology and Biotechnology in Western China, Ministry of Education, College of Life Sciences, Northwest University, Xi’an, Shaanxi Province 710069 P.R. China; 2 Department of Cell Biology, Duke University Medical Center, Durham, NC 27710 USA

## Abstract

The cytokinetic division ring of *Escherichia coli* comprises filaments of FtsZ tethered to the membrane by FtsA and ZipA. Previous results suggested that ZipA is a Z-ring stabilizer, since *in vitro* experiments it is shown that ZipA enhanced FtsZ assembly and caused the filaments to bundles. However, this function of ZipA has been challenged by recent studies. First, ZipA-induced FtsZ bundling was not significant at pH greater than 7. Second, some FtsA mutants, such as FtsA* were able to bypass the need of ZipA. We reinvestigated the interaction of FtsZ with ZipA *in vitro*. We found that ZipA not only stabilized and bundled straight filaments of FtsZ-GTP, but also stabilized the highly curved filaments and miniring structures formed by FtsZ-GDP. FtsA* had a similar stabilization of FtsZ-GDP minirings. Our results suggest that ZipA and FtsA* may contribute to constriction by stabilizing this miniring conformation.

## Introduction

FtsZ, a bacterial tubulin homologue, is the key cytoskeletal protein in the cytokinesis ring. In the presence of GTP, FtsZ monomers assemble into filaments, which associate further to form a ring around the middle of the cell. More than a dozen other cell division proteins assemble onto the FtsZ scaffold to form the divisome or Z ring, which constricts to divide the cell^1–3^. FtsZ filaments are tethered to the membrane by two membrane proteins, FtsA and ZipA in *E. coli*. FtsZ, FtsA and ZipA assemble into the proto-ring at the first stage of the division ring assembly^4–8^. FtsA binds a conserved peptide on the C-terminus of FtsZ, and has an amphipathic helix that inserts into the membrane^4–6^. ZipA is a transmembrane protein with a cytoplasmic domain that binds the same C-terminal peptide of FtsZ^6–8^. ZipA is an essential protein, but a hyperactive mutant of FtsA, FtsA*, can compensate for its absence^9^. Recently, a number of FtsA mutants have been identified that can bypass ZipA^10^.

In addition to its role in tethering FtsZ to the membrane, ZipA has been suggested to be a Z-ring stabilizer, since *in vitro* experiments it is shown that ZipA enhanced FtsZ assembly and caused the filaments to associate into bundles^11, 12^. However, the function of ZipA was challenged by recent studies. First, ZipA induced FtsZ bundling was not significant at pH greater than 7^13^. Second, as referenced above, some FtsA mutants are able to bypass the need of ZipA. The FtsZ mutant L169R can also bypass the need for ZipA^14^. Pichoff *et al*. suggest that FtsA* and other FtsA mutants decreased FtsA intrinsic self-interaction, thereby enhancing FtsA’s recruitment of downstream division proteins, which has been considered the role of ZipA^10, 15^. Third, ZipA is only found in γ-proteobacteria, and is therefore not a highly conserved member of the division machine.

In the present work, we have reinvestigated the interaction of FtsZ with ZipA *in vitro*. We found that ZipA stabilized not only the FtsZ-GTP filaments, but also the FtsZ-GDP miniring conformation. FtsA* had a similar stabilization of minirings. The miniring conformation of FtsZ-GDP has been suggested to generate the constriction force^3^. Our present work suggests that ZipA and FtsA* may contribute by stabilizing this miniring conformation and enhancing the constriction forces.

## Results

### FtsZ-ZipA assembles into filament bundles in the presence of GTP and into minirings in the presence of GDP

**Figure 1:**
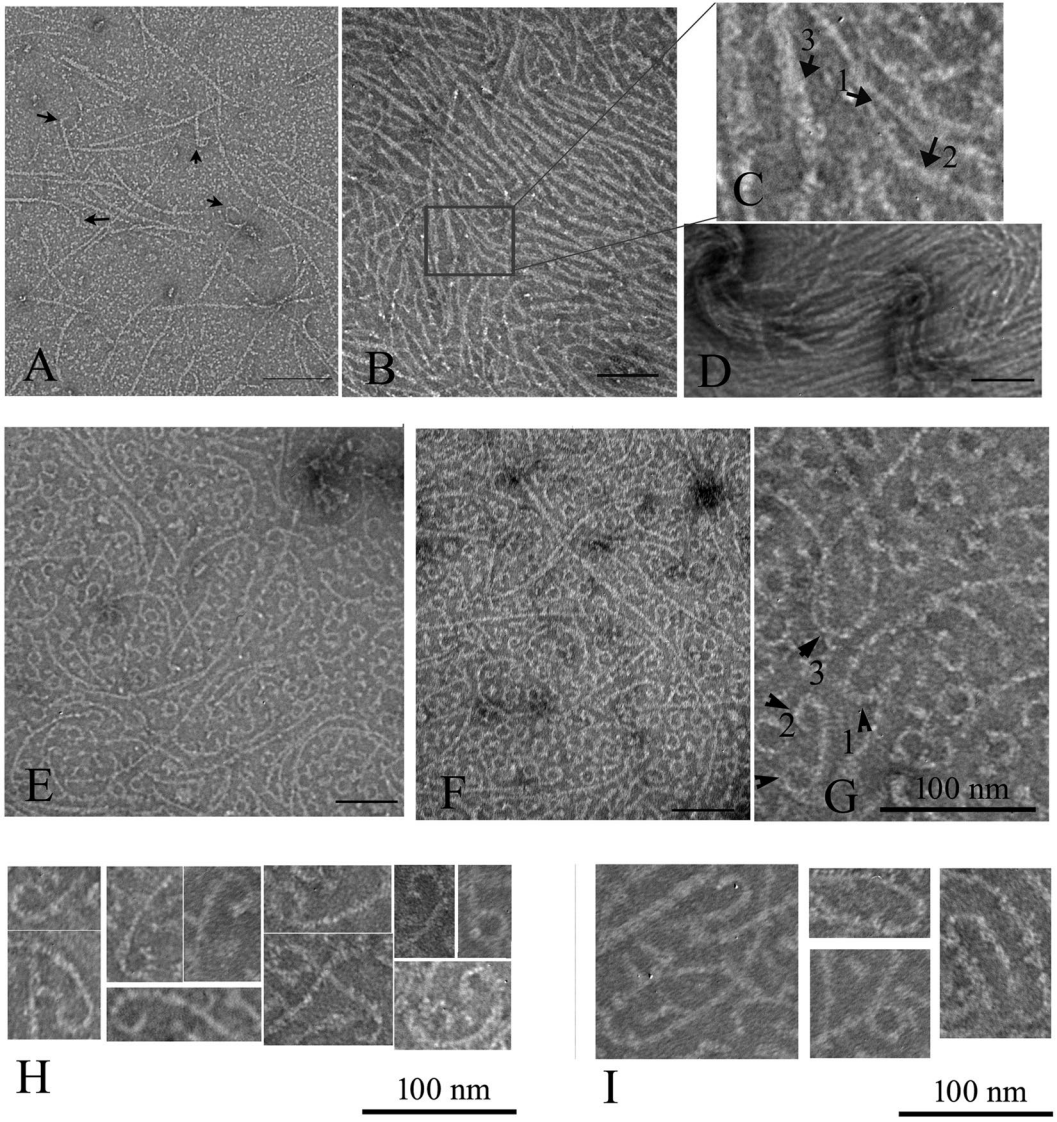
Electron microscopy (EM) images of FtsZ-ZipA at pH 6.5 and 7.5. (**A**) 5 µM FtsZ only, at pH 7.5. Polymers are mostly single straight filaments, with some short curved segments at the ends (arrows). (**B**–**E**) At pH 6.5, 5 µM FtsZ plus 5 µM ZipA assembles mostly into bundles (**B**–**D**). In (**C**) arrows identify FtsZ single filaments (arrow 1) and FtsZ-ZipA bundles of variable thickness (arrow 2 and 3). Some areas show single filaments mixed with highly curved filaments and minirings (**E**). (**F**–**G**) At pH 7.5, 5 µM FtsZ plus 5 µM ZipA assembles into single filaments and bundles, mixed with many highly curved filaments and minirings. The highly curved filaments locate mostly at the end of filaments (G, arrow 1), sometimes at both ends (**G**, arrow 2), or rarely in the middle of the filaments (**G**, arrow 3). (**H**–**I**) Selected EM images of the highly curved filaments at the end of filaments (**H**) or in the middle of filaments (**I**). Bars represent 100 nm.

Previous studies reported that ZipA enhanced FtsZ assembly in the presence of GTP^11, 12^. Our results confirmed this and further demonstrated how assembly varied with pH. Figure 1A shows the single, one-stranded protofilaments that are the typical assembly products of FtsZ alone. An equimolar mixture of FtsZ and ZipA, in MMK buffer at pH 6.5, assembled into wider filaments, which are probably sheets or bundles of protofilaments, since they sometimes fray into thinner protofilaments (Fig. 1B–D). In HMK buffer at pH 7.5, the sheets or bundles were thinner and single protofilaments were more pronounced (Fig. 1F). We concluded that there are three kinds of filaments after FtsZ assembled with the ZipA. The thin filaments (Fig. 1C, arrow 1) are the FtsZ filaments. The medium (Fig. 1C, arrow 2) and the thick filaments (Fig. 1C, arrow 3) are interpreted to be pairs and larger bundles of FtsZ filaments coated with ZipA.

Interestingly, FtsZ plus ZipA assembled into not only straight protofilaments, but also highly curved protofilaments and minirings (Fig. 1E–G). These minirings were occasionally seen at pH 6.5, and were much more abundant at pH 7.5. The minirings are similar to those previously obtained when FtsZ was assembled onto a positively charged lipid monolayer^16^. The miniring structures included both closed and partial circles, the latter located primarily at the end of the straight filaments. Rarely a curved segment was located in the middle of a straight filament (Fig. 1G,I). The partial minirings were mostly associated with thinner filament bundles.

**Figure 2:**
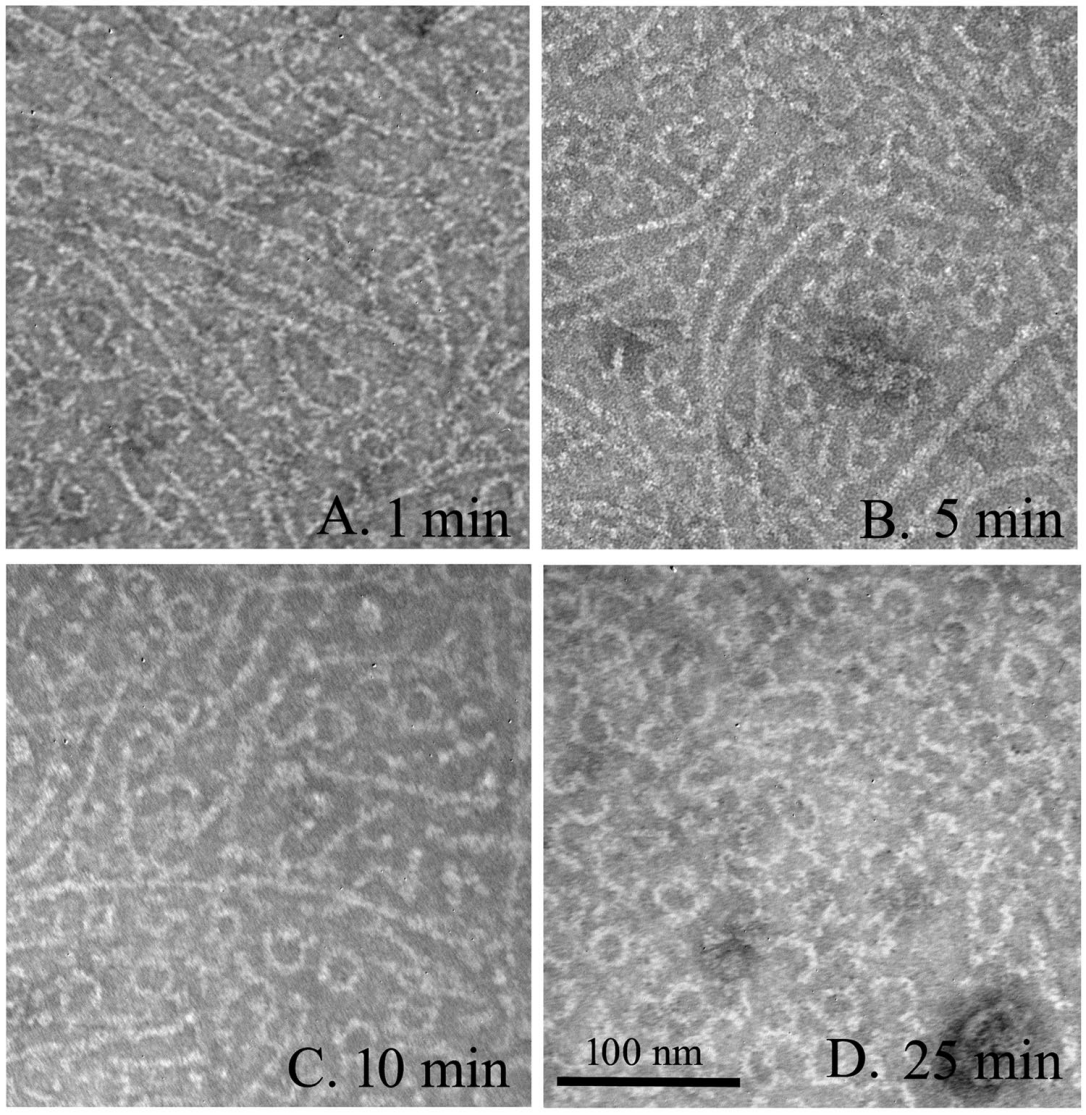
EM images of 5 µM FtsZ plus 5 µM ZipA, pH 7.5, at different times after adding 0.5 mM GTP. After 25 minutes, most filaments are miniring structures. Bar is 100 nm.

**Figure 3:**
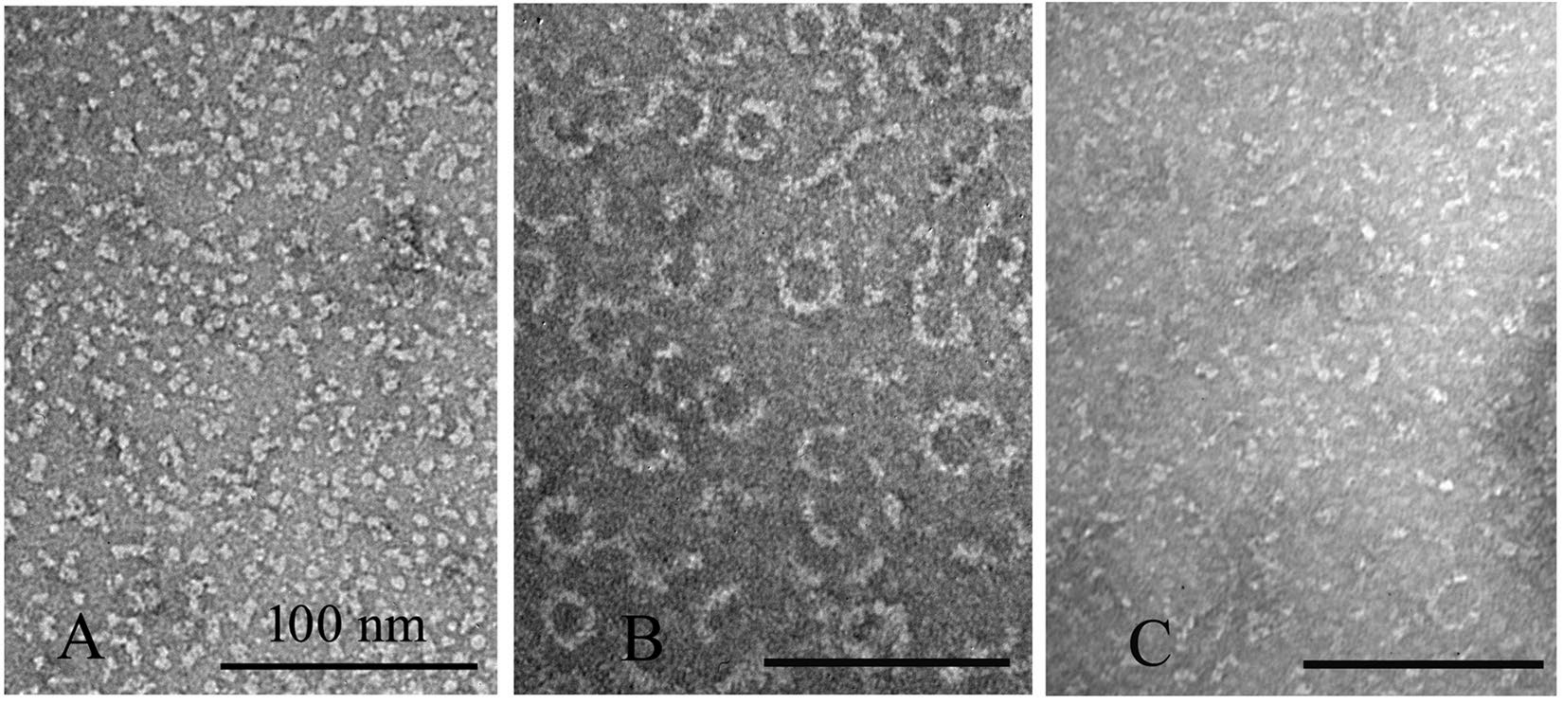
EM images of 5 µM FtsZ (**A**), 5 µM FtsZ plus 5 µM ZipA (**B**) and 15 µM FtsZ (**C**) assembled at pH 7.5 in 0.5 mM GDP. Bar is 100 nm.

To check whether these FtsZ-ZipA minirings related to GTP hydrolysis, we imaged samples at various times after adding 0.5 mM GTP to 5 μM FtsZ-ZipA (Fig. 2A–D). A few minirings appeared in the first minute after adding the GTP (Fig. 2A), and their number increased at 5 and 10 minutes (Fig. 2B,C). After 25 minutes, most straight protofilaments had disappeared and minirings predominated (Fig. 2D). To confirm that GDP favored the minirings, we initiated assembly by adding 0.5 mM GDP. This produced abundant minirings that were mostly closed and good candidates for measurement (Fig. 3B). The outside diameters of 30 minirings gave an average and standard deviation of 25.2 ± 3.1 nm, and a thickness of 5.2 ± 0.7 nm. This is similar to the 23 nm diameter measured for minirings assembled onto the charged lipid monolayers^16^. The increased diameter may be due to the ZipA, which, although not resolved, presumably accompanies FtsZ in these Z rings. Without ZipA, FtsZ usually assembles into some oligomers in the presence of GDP (Fig. 3A), but several minirings or partial minirings are observed randomly if FtsZ concentration increases to 15 μM (Fig. 3C).

**Figure 4:**
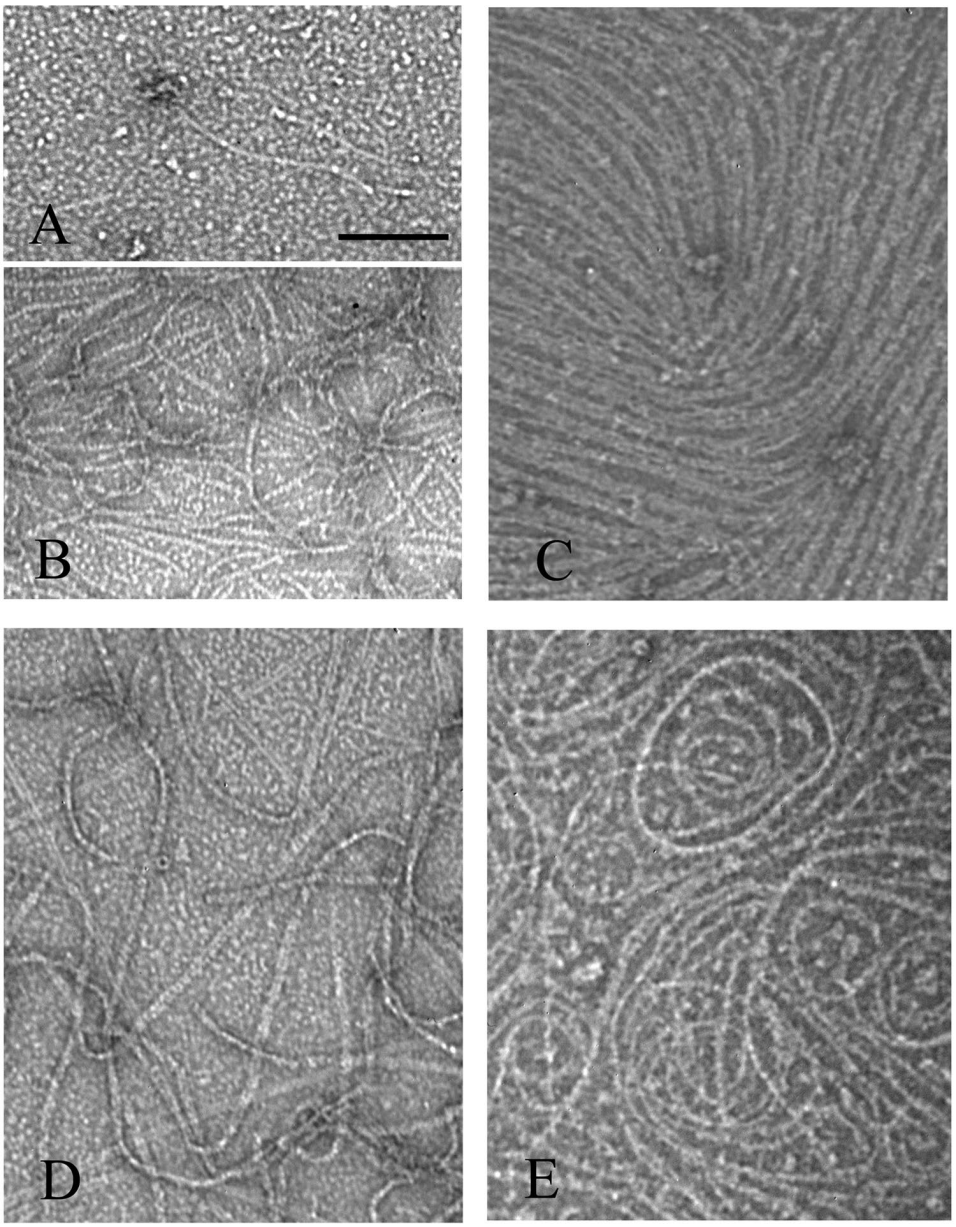
EM images FtsZ and FtsZ-ZipA assembled with EDTA and GTP, pH 6.5 (**A**–**C**), or in HMK pH 7.5 with GMPCPP (**D**,**E**). In MEK buffer (50 mM MES, pH 6.5, 100 mM KAc, 1 mM EDTA) plus GTP, a few filaments were assembled with 5 µM FtsZ (**A**) and more with 10 µM FtsZ (**B**). 5 µM FtsZ plus 5 µM ZipA in MEK assembled abundant thick filament bundles (**C**). In HMK buffer, pH 7.5, in 0.5 mM GMPCPP, 5 µM FtsZ assembled into long filaments and bundles (**D**). 5 µM FtsZ plus 5 µM ZipA in GMPCPP, pH 7.5, assembled into long filaments with a tendency to curve (**E**). Bar represents 100 nm.

We also checked the effects of ZipA on FtsZ assembly in EDTA buffer, which blocks GTP hydrolysis, and in GMPCPP (Guanosine-5′-[(α,β)-methyleno]triphosphate), which is a slowly hydrolysable GTP analog. In MEK buffer (50 mM MES, pH 6.5, 100 mM KAc, 1 mM EDTA), 5 μM FtsZ produced few filaments (Fig. 4A), but 10 μM FtsZ assembled abundant long filaments (Fig. 4B). This is consistent with the high critical concentration for assembly in EDTA^17^. The addition of 5 µM ZipA to 5 µM FtsZ greatly enhanced assembly, producing thick filaments and sheets (Fig. 4C). Assembly of 5 µM FtsZ in GMPCPP in HMK buffer (pH 7.5) produced very long filaments and bundles (Fig. 4D). The addition of ZipA to the GMPCPP assembly increased the assembly and favored curvature with diameters from 100 nm to 200 nm. This is similar to the intermediate curved conformation, which can be obtained without GTP hydrolysis^3^. No minirings were formed in either EDTA-GTP or Mg-GMPCPP, consistent with the interpretation that minirings are favored by GDP.

### FtsZ-FtsA* also assembles into minirings in GDP and bundles in GTP

**Figure 5:**
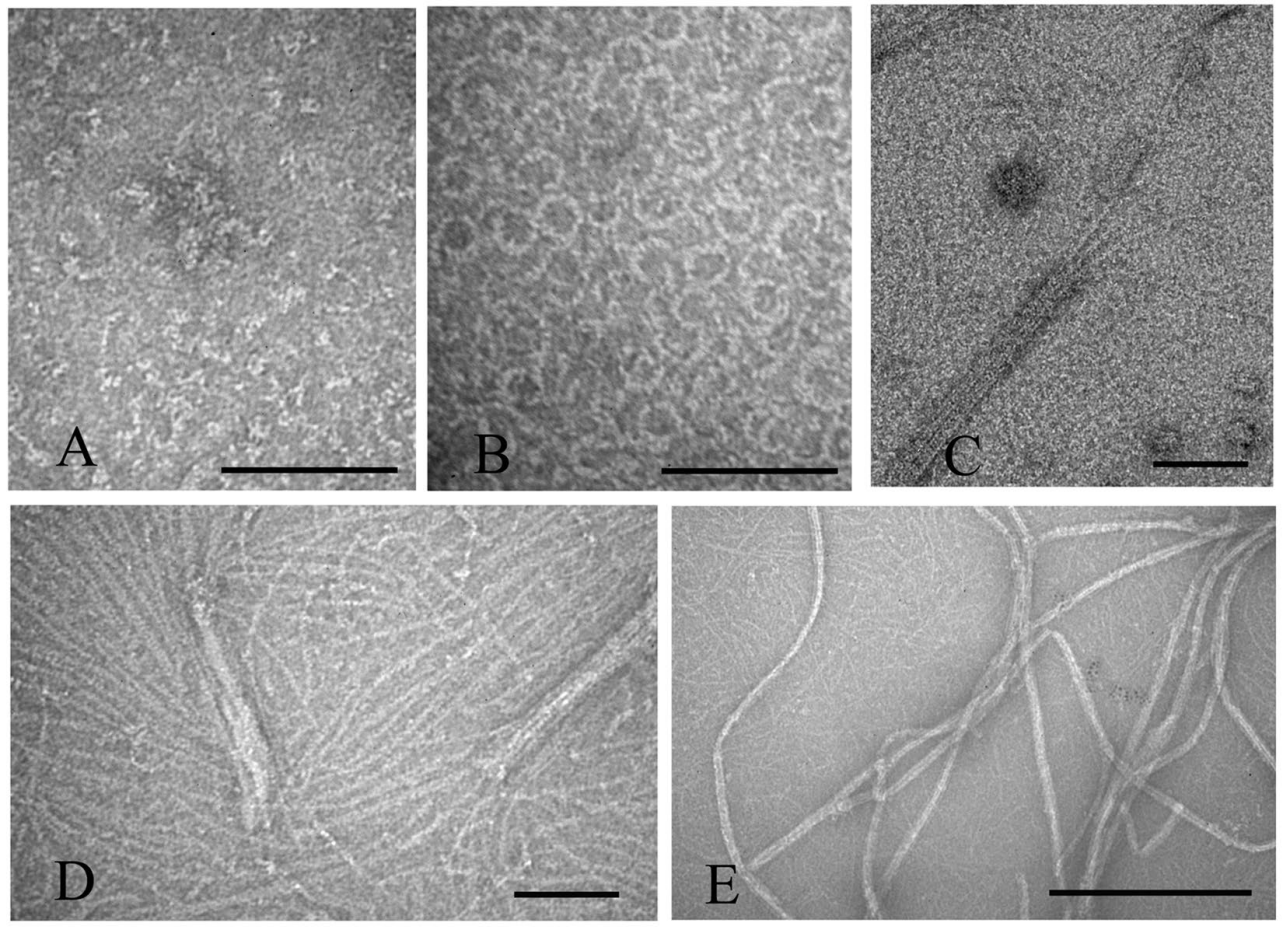
Assembly of FtsZ plus FtsA or FtsA*, at pH 7.5. (**A**,**B**) Assembly in 0.5 mM GDP of 15 µM FtsZ plus 10 µM FtsA (**A**) or 10 µM FtsA* (**B**). FtsA* caused assembly of abundant minirings. (**C**–**E**) Assembly in 1 mM GTP of 10 µM FtsZ plus 10 µM FtsA* without ATP (**C**,**D**), and with 1 mM ATP (**E**). Bars are 100 nm in **A**–**D** and 500 nm in **E**.

FtsA is another FtsZ interacting protein that can tether FtsZ to the membrane. Recently studies have shown that an FtsA mutant, FtsA* (FtsA R286W) can bypass the need of ZipA^9^. We prepared purified FtsA and FtsA* to test their effects on FtsZ assembly. For assembly in GDP, FtsA had minimal effect, whereas FtsA* strongly enhanced assembly of minirings (Fig. 5A,B). The FtsZ-FtsA* minirings had an outside diameter of 21.9 ± 2.5 nm (n = 25), somewhat smaller than the 25.2 ± 3.1 nm of FtsZ-ZipA minirings.

FtsZ assembly in GTP was also affected by FtsA*, which caused some protofilaments to coalesce into large bundles, mixed with smaller protofilaments and bundles (Fig. 5C,D). When 1 mM ATP was added to the assembly, the bundling was greatly enhanced (Fig. 5E). Our FtsA protein had no effect on this assembly either, suggesting that it may be inactive.

### Dynamic properties of FtsZ-ZipA and FtsZ-FtsA

FtsZ-ZipA and FtsZ-FtsA* assembled into bundles, which might have altered the kinetics of assembly and the rates of subunit exchange at steady state. The GTPase activity showed no significant difference for FtsZ alone and with added ZipA or FtsA* (measured three times), consistent with the previous studies^11, 12, 18^. This suggests that subunit exchange into protofilaments was not substantially changed by the bundling. We extended this observation with our range of fluorescent assays. Our ATTO-fluorescence quenching gives a quantitative assay of polymer, specifically the number of protofilament interfaces, independent of bundling^19, 20^. Light scattering, on the other hand, is highly sensitive to bundling. Finally, our FRET assay measures the exchange of subunits upon mixing pre-assembled protofilaments that were labeled separately with fluorescein and rhodamine^21^.

**Figure 6:**
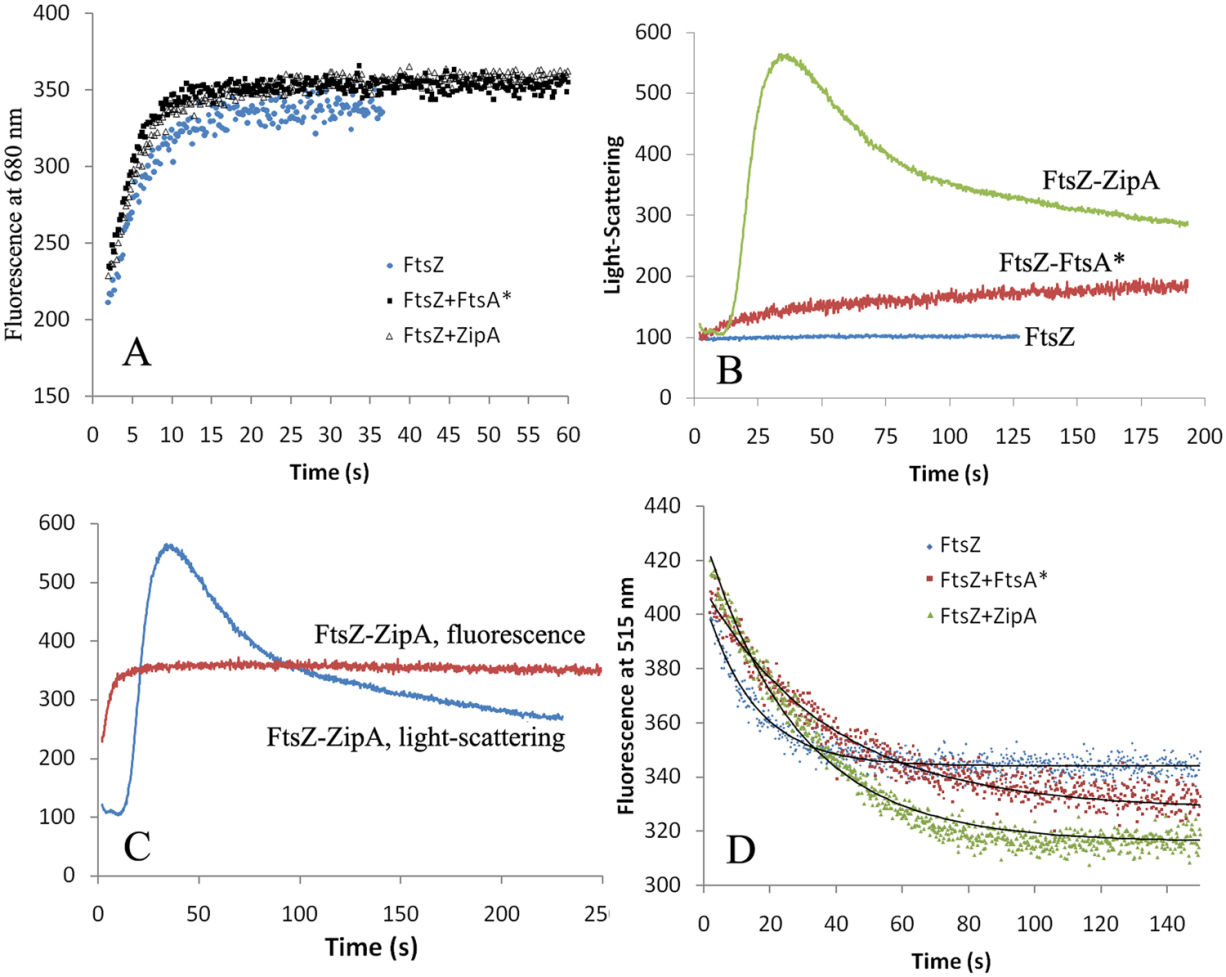
Dynamics of the FtsZ assembly and filament turn-over in MMK buffer, pH 6.5. (**A**) FtsZ assembly kinetics measured by Atto fluorescence. Assembly is almost the same for FtsZ alone and with ZipA and FtsA*. (**B**) FtsZ assembly kinetics measured by light-scattering. The enhanced light scattering with ZipA and FtsA* reflects bundling. (**C**) Direct comparison of the assembly kinetics of FtsZ-ZipA mixture measured by Atto fluorescence and light-scattering. Filament assembly reported by ATTO fluorescence precedes the bundling reported by light scattering. (**D**) The kinetics of FtsZ filament turn-over measured by FRET technique. The turn-over rates are slower for polymers with ZipA or FtsA*.

For the ATTO quenching assay, we mixed 1 part labeled FtsZ with 9 parts unlabeled, so that the kinetics are dominated by the unlabeled protein. Figure 6A shows that assembly of FtsZ rose rapidly to a plateau in ~10 s. The addition of ZipA or FtsA* caused a slight acceleration in assembly and did not alter the plateau. Figure 6B shows the light scattering assay. FtsZ alone gave virtually no light scattering signal, consistent with the small scattering profile for the one-subunit wide protofilaments. Assembly with FtsA* produced a modest light scattering over the first 25 s, followed by a slow, continuous rise. Assembly with ZipA showed a 10–15 s lag in light scattering followed by a strong rise to a peak at 30 s, and a decline over the next few minutes. The light scattering only began to rise after the ATTO fluorescence had plateaued (Fig. 6C), consistent with rapid assembly of single protofilaments followed by their association into bundles. Finally, we used the FRET (Fluorescence resonance energy transfer) assay to measure the rate of subunit exchange for protofilaments at steady state. The data for all three reactions were fit well by a single exponential, $\textcolor[rgb]{0,0,0}{\text{starF}}(t)=\,Fo+a\ast {e}^{-t/\tau }$. The reaction time τ was 13.1 s for FtsZ alone, 25.2 s for FtsZ-ZipA and 38.0 s for FtsZ-FtsA* in the pH 6.5 buffer (Fig. 6D).

### PaZipA (ZipA from *Pseudomonas aeruginosa*) also enhances and stabilizes PaFtsZ (FtsZ from *P. aeruginosa*) minirings

We purified full-length PaFtsZ and the cytoplasmic domain of PaZipA to test whether PaZipA would stabilize the miniring conformation. The globular core of *E. coli* FtsZ (residues 10–316) shows 67% amino acid identity with PaFtsZ. The globular FtsZ-binding domain of PaZipA shows 40% identity to the *E. coli* sequence.

**Figure 7:**
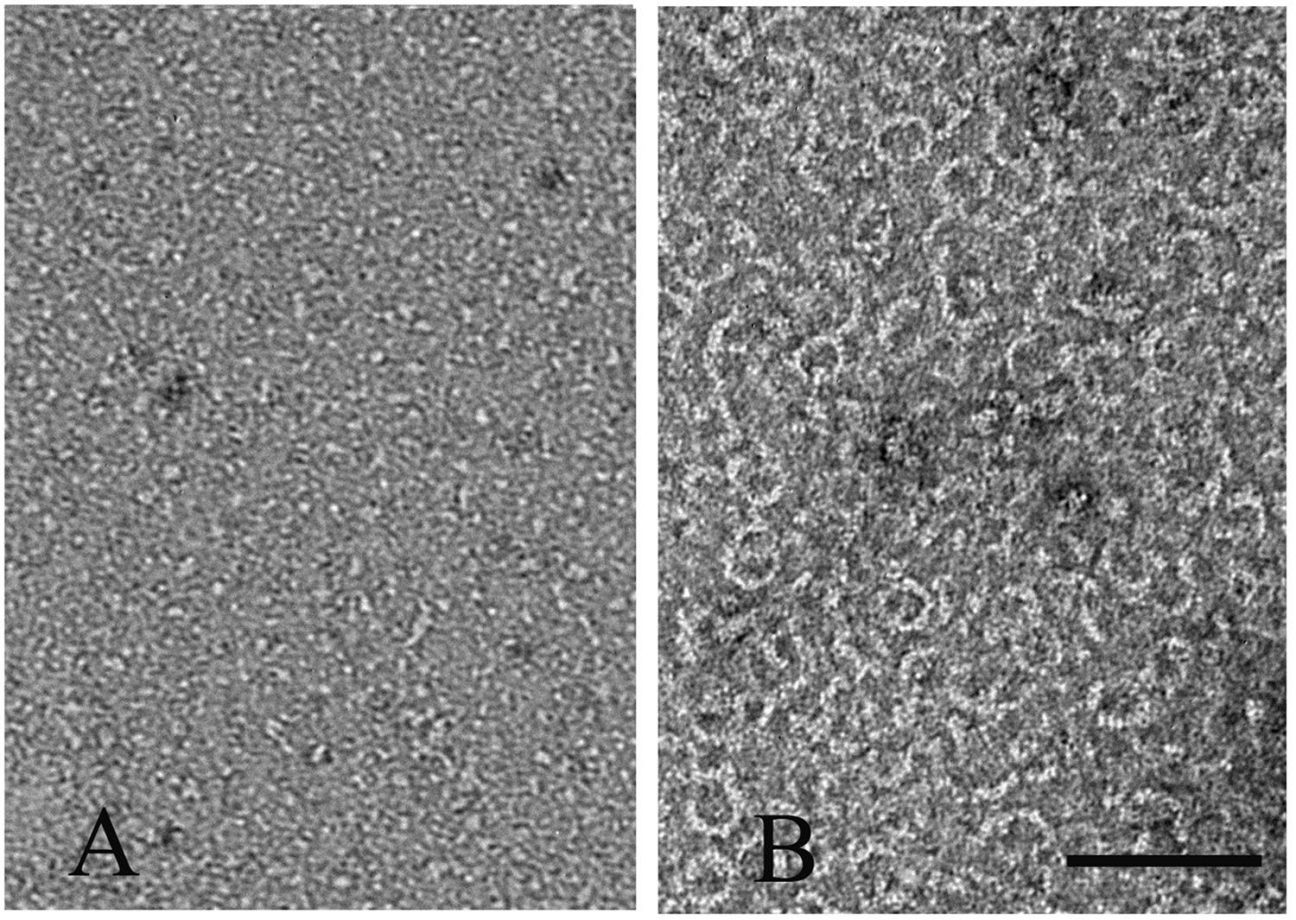
EM images of 5 µM PaFtsZ alone (**A**) and with 5 µM PaFtsZ-ZipA (**B**) in 0.5 mM GDP. The buffer is HMK, pH 7.5. Bar is 100 nm.

Figure 7 shows the electron microscopy (EM) images of PaFtsZ alone and with PaZipA in the presence of GDP. PaFtsZ alone assembled only a few short oligomers (Fig. 7A). However, the equimolar mixture of PaFtsZ and PaZipA assembled abundant curved polymers (Fig. 7B). Many of these were partial rings, but some formed closed minirings very similar to the *E. coli* proteins.

## Discussion

Both ZipA and FtsA are membrane binding proteins and are important for *E. coli* cell division. Since ZipA only exists in γ-proteobacteria and mutations in FtsA or FtsZ could by-pass the need of the ZipA^9, 10, 14, 15, 22^, the role of the ZipA is controversial. To obtain more information about ZipA function, we compared the biochemical properties of the ZipA and FtsA* effects on the FtsZ assembly.

Previous reports suggested that the ZipA enhanced FtsZ assembly into bundles *in vitro* and thus stabilized the Z-ring structure *in vivo* ^11, 12, 23^. Our results confirm this and suggest that FtsZ-ZipA assembles first into single filaments, similar to FtsZ alone, which then associate laterally to form sheets or bundles. The ZipA is not resolved but presumably bridges the FtsZ filaments. Bundle formation of the FtsZ-ZipA mixture occurred primarily below pH 7. At pH 7.5, which is close to the physiological condition, FtsZ alone assembled into mostly single protofilaments, consistent with previous results^13^. ZipA enhanced filament bundling somewhat at pH 7.5, and also stabilized minirings and highly curved filaments.

The simplest explanation for GTP hydrolysis at steady state postulates that for each hydrolysis event an FtsZ-GDP subunit must dissociate from the polymer, exchange GDP for GTP, and re-polymerize. In this case, the GTPase should equal the rate of exchange into the polymers. However, we found that the subunit exchange was slower in ZipA, whereas the GTPase was unchanged. We have previously observed that there is also exchange independent of GTP hydrolysis^24^. This may be the main contribution to the slower exchange in ZipA.

We found that ZipA not only enhanced FtsZ assembly in GTP, but also stabilized the FtsZ polymers in GDP. FtsZ-GDP polymers are highly curved filaments that tend to form minirings. Without ZipA, FtsZ-GDP polymers were seen only rarely at high concentrations of FtsZ (15 µM), consistent with a previous report^25^. In contrast, 5 µM FtsZ plus 5 µM ZipA in GDP assembled abundant curved filaments and minirings. A few minirings can be seen in the negative EM image of Mateos-Gil *et al*.^13^, although these authors did not remark on them.

Minirings were observed previously when FtsZ-GDP was assembled on cationic lipid monolayers^16^. Helical tubes were assembled by FtsZ-GDP when stabilized by DEAE dextran^26, 27^. That led to the suggestion that the curved conformation in solution was not a planar circle, but a helix with a pitch. The assembly of minirings with ZipA suggests that the ZipA tends to reduce the pitch so that the curved filaments can form closed circles in solution. Similar to the original description of minirings^16^, they are sometimes seen as a highly curved segment at one end of a straight filament. Rarely are they seen at both ends or in the middle.

FtsA*, a hyperactive FtsA mutant that could bypass the need of ZipA^9^, also stabilized miniring assembly by FtsZ-GDP. Wild-type FtsA had no activity. This is consistent with the lack of activity in previous studies, and the suggestion that the wild type FtsA is difficult to be expressed and purified^28^. Assembly of FtsZ plus FtsA* in GTP gave enhanced bundles of straight filaments. Upon addition of ATP, the bundling was further enhanced. In a previous study, Beuria *et al*.^18^ found that FtsZ + FtsA* + GTP + ATP produced bundles of filaments with a well-defined curvature of ~200 nm diameter. We have referred to this as the “intermediate curved conformation”^3, 29^. Our micrographs showed some tendency for curvature, but were mostly straight. We have not yet determined what assembly conditions would give the enhanced curvature seen by Beuria *et al*.^18^.

We have suggested previously that the transition from straight FtsZ filaments to the highly curved miniring conformation might generate the force for constriction of the Z ring^3^. The present work shows that two proteins closely associated with FtsZ, ZipA and FtsA*, enhance and stabilize the miniring conformation, and therefore may contribute to the constriction force. In our studies, we found that ZipA from both *E. coli* and *P. aeruginosa* have similar functions. Since ZipA is only found in γ-proteobacteria, there might be other proteins in other bacteria with similar functions. One example might be FzlA from *Caulobacter crescentus*, which caused FtsZ to form miniring structures in GDP and assemble into straight filaments in GTP^30^.

Recent work has suggested that constriction mechanism is more complex than a simple curved filament bending membranes. Coltharp *et al*.^31^ showed that the rate of constriction was limited by cell wall synthesis, and suggested that this synthesis might provide the major constriction force. Two groups have shown that FtsZ is organized in patches that move around the Z ring by FtsZ treadmilling^32, 33^. Cell wall synthesis also occurs in patches that presumably follow the FtsZ. Bisson-Filho *et al*.^33^ suggested that treadmilling FtsZ might generate an initial small constriction, which is reinforced by the following cell wall synthesis. Future work will be needed to integrate the FtsZ curved conformation into these moving patches and associated cell wall synthesis.

## Methods

### Protein Purification

Expression vectors for *E. coli* FtsZ and mutants (FtsZF268C, FtsZY222W/T151C) were expressed from the plasmid pET11b and were purified as described previously^19, 21^. Briefly, the over-expressed FtsZ proteins were precipitated by 30% saturated ammonium sulfate, followed by chromatography on a source Q 10/10 column (GE healthcare) with a linear gradient of 50–500 mM KCl in 50 mM Tris, pH 7.9, 1 mM EDTA, 10% glycerol. Peak fractions were identified by SDS-PAGE and stored at −80 °C.

N-terminally truncated ZipA (26–328) from *E. coli* was inserted into the plasmid pET15b at the NdeI/BamHI sites. The soluble His6-ZipA protein was purified by affinity chromatography on a Talon column (Clontech Lab, Inc.). The His-tag was removed by incubating with 2 units/ml of thrombin.

FtsA and FtsA-R286W (FtsA*) from *E. coli* were purified as described^28^. Briefly, following expression, bacteria were suspended in lysis buffer (50 mM Tris HCl, pH 7.9, 350 mM KCl, 10% glycerol), sonicated and centrifuged for 20 min at 32,000 rpm (Beckman rotor Ti41.2); the expressed His6-tag protein was mostly in the pellet. The pellet was suspended in lysis buffer containing 1% Triton X-100. The suspension was centrifuged for 20 min again at 32,000 rpm, and the expressed protein now remained mostly in the supernatant. The soluble protein was applied to a Talon column and eluted with buffer containing 150 mM imidazole. DTT was added to 0.1%, and the protein was stored at −80 °C.

Expression vectors for PaFtsZ and N-terminal truncated PaZipA were constructed in the plasmid pET15b at the NdeI/BamHI sites. The soluble proteins were purified by affinity chromatography on a Talon column (Clontech Lab, Inc.). The purified His6-PaFtsZ was incubated with 2 units/ml of thrombin for 2 hours at room temperature to remove the His-tag. PaFtsZ was further purified by chromatography on a source Q 10/10 column (GE healthcare) with a linear gradient of 50–500 mM KCl in 50 mM Tris, pH 7.9, 1 mM EDTA and 10% glycerol. Proteins were stored at −80 °C.

### Assays of FtsZ assembly kinetics and filament turn-over

FtsZ assembly kinetics were measured using three assays: FRET (Fluorescence resonance energy transfer), Trp quenching of ATTO fluorescence and light-scattering, as described previously^19, 21, 24^. For the FRET assay, a single cysteine mutant of FtsZ (FtsZ-F268C) was labeled separately with Fluorescein 5-maleimide (Molecular Probes) as donor and Tetramethylrhodamine 5-maleimide (Molecular Probes) as acceptor. FtsZ assembly was tracked using the decrease in donor fluorescence at 515 nm, with excitation at 470 nm as described previously^21^. For the ATTO-trp quenching assay, a double mutant FtsZT151C/Y222W was labeled with ATTO-655-maleimide (Fluka). For assembly experiments, the labeled FtsZ protein was diluted with a 9-fold excess of unlabeled wild-type protein to avoid the formation of FtsZ bundles^19^. ATTO fluorescence spectra were measured at the ATTO emission peak 680 nm, with excitation at 650 nm as described previously^19^. For the light-scattering assay, the fluorometer was used with both excitation and emission at 340 nm. Fluorescence measurements were taken with a Shimadzu RF-5301 PC spectrofluorometer in a thermostatically controlled cell at 25 °C.

Assembly kinetics were mostly measured in two buffers. HMK buffer contains 50 mM HEPES (pH 7.5), 100 mM KAc, 5 mM MgAc; MMK buffer contains 50 mM MES (pH 6.5), 100 mM KAc, 5 mM MgAc.

### Electron Microscopy

Negative stain electron microscopy was used to visualize FtsZ filaments, as described previously^34^. FtsZ was incubated with GTP or GDP for several minutes and ~10 µl was applied to a UV treated carbon-coated copper grid and quickly withdrawn. Grids were immediately stained with 2% uranyl acetate, and specimens were imaged with a Philips 420 electron microscope. Images were recorded on a CCD camera at a nominal magnification of 49,000 to 82,000.

FtsZ or mixtures of FtsZ plus ZipA or FtsA were assembled with GTP, GDP (Sigma-Aldrich) and GMPCPP (a gift from Dr. Michael Caplow, University of North Carolina).
